# Characteristics of sleep structure in Parkinson's disease patients with hallucinations based on polysomnography

**DOI:** 10.3389/fneur.2022.929569

**Published:** 2022-11-01

**Authors:** Ruxin Gu, Jun Zhu, Min Zhong, Yinyin Jiang, Sha Zhu, Yaxi Wang, Xu Jiang, Bo Shen, Jun Yan, Li Zhang, Yang Pan

**Affiliations:** ^1^Department of Geriatric Neurology, Nanjing Brain Hospital Affiliated to Nanjing Medical University, Nanjing, China; ^2^Institute of Neuropsychiatric Diseases, Nanjing Brain Hospital Affiliated to Nanjing Medical University, Nanjing, China

**Keywords:** Parkinson's disease, hallucination, sleep disorders, slow wave sleep, sleep quality

## Abstract

Hallucination is a common non-motor symptom in patients with Parkinson's disease (PD). Additionally, sleep disorders are associated with an increased risk of hallucinations in PD patients. This study aimed to examine the association between hallucination and objective sleep parameters in PD patients. We retrospectively recruited 278 PD patients who underwent polysomnography and clinical assessments and classified them into non-hallucination and hallucination groups. Hallucinations were observed in 77 older PD patients who had more severe motor symptoms and higher scores on the Non-Motor Symptoms Questionnaire (NMSQ), Hamilton Depression Scale (HAMD) and Hamilton Anxiety Scale (HAMA) but lower scores on the Montreal Cognitive Assessment (MOCA) and PD Sleep Scale (PDSS) than PD patients without hallucinations. Analysis of the polysomnographic variables in patients with hallucinations showed that they exhibited a decrease in total sleep time, sleep efficiency (SE), rapid eye movement (REM) sleep time and slow wave sleep (SWS, N3) time and percentage but a significant increase in wake time after sleep onset (WASO), periodic limb movement index (PLMI) scores, and stage 2 NREM (N2)percentage. Logistic regression analysis revealed that higher NMSQ scores, lower MOCA scores, lower SE, and a lower percentage of N3 sleep were associated with hallucinations in PD patients. Our results suggested that PD patients with hallucinations had worse sleep quality and differences in sleep architecture (measured by polysomnography).

## Introduction

Except typical motor deficits, Parkinson's disease (PD) patients suffer from a range of non-motor symptoms (NMS), such as Parkinson's disease psychosis (PDP), sleep disturbances, cognitive deficits, and autonomic dysfunction (1). With disease progression, NMS predominate, reducing quality of life; this is especially true for PDP and sleep disturbances, which are associated with higher mortality and greater rates of nursing home placement (2, 3).

Approximately 2/3 of patients with PD experience hallucinations during their disease course (4). Most patients with PDP present with minor hallucinations (MH) and well-structured visual hallucinations (VH), which may precede motor symptoms (5).

Sleep disturbances are common in PD, can present in prodromal stages, such as REM sleep behavior disorder (RBD) (6). Moreover, RBD may increase the risk of hallucinations (7).

Several studies (8) have suggested that PD patients who developed hallucinations had worse sleep quality than those who did not, with reduced sleep duration, reduced efficiency and increased daytime sleepiness. This is consistent with evidence reporting that poor sleep quality played a causal role in the development of psychotic experiences, such as auditory hallucinations and VH (9). In addition, imaging data from PD patients with hallucinations or sleep disturbances showed dysfunction of the cholinergic system in the brain; both groups of patients exhibited similar neurobiological processes of cholinergic dysfunction (10). These evidences indicate that sleep disorders associated with hallucinations, but few studies have accurately quantified sleep architecture to determine the association between hallucinations and sleep disorders.

Therefore, we aimed to examine and compare clinical characteristics and sleep parameters in a cohort of PD patients with or without hallucinations.

## Methods

### Patients

From January 2020 to March 2022, PD patients were recruited from the Affiliated Brain Hospital of Nanjing Medical University. This cross-sectional study evaluated their sleep disturbances. All participants met the Movement Disorder Society PD Criteria (11). Data from 18 patients who did not cooperate with sleep monitoring were excluded, resulting in the inclusion of 278 PD patients (Supplementary Figure S1). This study was approved by the Ethics Committee of the Affiliated Brain Hospital of Nanjing Medical University and written informed consent was obtained from all participants. We excluded patients with secondary Parkinsonism, focal brain injury (MRI evidence), severe ocular disorders and other psychiatric disorders. The patients did not take antipsychotics and hypnotics. Patients were also excluded if they had cognitive impairment, as demonstrated by a Montreal Cognitive Assessment (MoCA) score < 20 (12).

### Clinical assessment

Structured interviews were conducted using a standard questionnaire to obtain demographic characteristics and specific disease-related data, including sex, age, body mass index (BMI), duration of disease, and medication use. The questionnaire was administered by trained experts during face-to-face interviews. Levodopa equivalent doses (LEDs) were calculated according to previously published recommendations (13).

Motor symptoms were assessed using the Unified Parkinson's Disease Rating Scale motor subscale (UPDRS-III) and the Hoehn and Yahr scale (HY). Non-motor symptoms were assessed using the Non-Motor Symptom Questionnaire (NMSQ) (14), excluding the score on the hallucinations/delusions domain. Cognitive function was assessed according to the MOCA, which is more sensitive than the Mini-Mental State Examination (MMSE) for detecting mild cognitive impairment (15). The PD Sleep Scale (PDSS), Hamilton Anxiety Scale (HAMA) and Hamilton Depression Scale (HAMD) were used to assess subjective sleep quality and the severity of anxiety and depression, respectively (16–18).

The presence of hallucinations was assessed based on the “Hallucinations and Psychosis” item of the Movement Disorder Society-sponsored revision of the Unified Parkinson's Disease Rating Scale (MDS-UPDRS): 0: Normal = No hallucinations or psychotic behavior. 1: Slight = Illusions or non-formed hallucinations, but the patient recognizes them without loss of insight. 2: Mild = Formed hallucinations independent of environmental stimuli. No loss of insight. 3: Moderate = Formed hallucinations with loss of insight. 4: Severe = Patients have delusions or paranoid. A total of 201 patients were categorized into the non-hallucinations group. A questionnaire comprising 11 items on the nature and properties of patient hallucinations, including temporal factors, content and external factors, was used to further assessed hallucinations in patients with MDS-UPDRS hallucination scores ≥ 1 (19).

### Objective sleep assessment

Standard PSG assessment was performed at both sites using a video-enabled Compumedics Grael-HD 64 (Compumedics Grael-HD, Australia) polygraph throughout the night. The time of the PSG recording was fixed (between 23:00 and 07:00). We collected the following channels for sleep staging: 6 electroencephalogram channels (F3/M2, F4/M1, C3/M2, C4/M1, O1/M2, O2/M1); 2 electrooculogram channels (E1, E2), an electromyogram channel, a nasal pressure channel, a respiratory thermistor channel, an oximetry channel, a snoring sensor channel, an electrocardiography channel, a thoracic and abdominal respiratory effort sensor, a body position channel and 2 leg movement sensors.

Movements, sleep stages and cardiopulmonary events were assessed manually according to the American Academy of Sleep Medicine (AASM) guidelines (20), including periodic limb movement index (PLMI), five sleep stages (wake, stage N1, stage N2, stage N3, and REM), micro-arousal index (MAI) and apnea hypopnea index (AHI). Total sleep time (TST), wake time after sleep onset (WASO), sleep efficiency (SE), sleep latency (SL), and REM sleep latency (RSL) were recorded. Sleep disorders were determined for WASO ≥ 60 min, SL ≥ 30 min, and SE < 85% on the PSG as well as PDSS < 105 (21).

### Statistical analysis

Descriptive data are expressed as the mean ± standard deviation (SD) and number (n) or percentage (%). The two groups were compared, using Student's *t* test for continuous variables and the chi-square test for categorical variables. Clinical scores (HAMA, HAMD, MOCA, NMSQ, PDSS and PSG data) were compared by one-way analyses of covariance (ANCOVA) with adjustments for confounding factors, including age, UPDRS-III score and HY stage. A binary logistic regression model was used to identify possible factors associated with hallucinations. Receiver operating characteristic (ROC) curves were plotted to evaluate the abilities of the NMSQ, MoCA and 2 sleep variables to predict PD with hallucinations. Statistical analyses were performed using SPSS 27.0 (IBM Corporation, New York, USA), and a *p* < 0.05 value was considered statistically significant.

## Results

### Comparisons of motor symptoms and demographics between the PD patients with and without hallucinations

The final analysis included data from of 278 patients consisted of 112 females (40.3%) and 166 males (59.7%), with a mean age of 66.80 ± 8.64 years. Among these patients, 77 (25.8%) were diagnosed with hallucinations. Almost all of the patients who had hallucinations experienced minor hallucinations and/or well-structured visual hallucinations, sometimes with difficulty in distinguishing between the two. Some patients also reported other forms of hallucinations: 3 had tactile hallucinations, 4 had olfactory hallucinations, and 9 had auditory hallucinations (Table 1). Patients reported that hallucinations could be more likely to occur at night or in dim lighting (75.3%). The visual hallucinations tended to consist of mundane content, including unfamiliar or deceased people, animals, and objects. The following were significantly associated with hallucinations in the univariate analyses (Table 2): older age (*P* = 0.014, *P* < 0.05), higher UPDRS-III scores (*P* = 0.002, *P* < 0.05), and greater HY stage (*P* < 0.001). There were no differences in sex, BMI or drug types between the patients with hallucinations and those without. Although patients with hallucinations had a longer duration of disease and higher LEDs, these differences were not significant.

**Table 1:** Characteristics and contents of hallucinations.

**Characteristics**	**Number**	**%**
**Severity**		
Stage I	55	71.4%
Stage II	16	20.8%
Stage III	6	7.8%
**Content**		
People	47	61.0%
Animals	16	20.8%
Objects or cannot describe	14	18.2%
**Frequency**		
Daily	21	27.3%
1–6 times/week	28	36.4%
< 1/week	28	36.4%
**Lasting time**		
Seconds	50	64.9%
Minutes	27	35.1%
**Color**		
Black and white	31	40.3%
Single color	22	28.6%
Multiple colors	24	31.2%
**Lighting**		
Bright	9	11.7%
Dim	36	46.8%
Dark	22	28.6%
Variable	10	13.0%
**Movement**		
Mobile	57	74.0%
Still	20	26.0%
**Stereotyped**		
Yes	45	58.4%
No	32	41.6%
**Size**		
Normal	51	66.2%
Miniaturized	15	19.5%
Magnified	11	14.3%
**Clarity**		
Sharp	24	31.2%
Blurry	43	55.8%
Variable	10	13.0%
**Other forms of hallucinations**		
Auditory	9	11.7%
Olfactory	4	5.2%
Tactile	3	3.9%

**Table 2:** Demographic and clinical characteristics of Parkinson's disease patients based on the presence/absence of hallucinations.

	**Total**	**With hallucinations**	**Without hallucinations**	***P***
*n*	278	77 (25.8%)	201 (74.2%)	
Age, y^a^	66.80 ± 8.64	68.84 ± 8.09	66.01 ± 8.74	0.014*
Male sex, *n* (%)^b^	166 (59.7%)	46 (59.7%)	120 (59.7%)	0.995
BMI^a^	23.66 ± 3.31	23.55 ± 2.79	23.71 ± 3.49	0.735
Duration, y^a^	6.19 ± 4.21	6.82 ± 4.58	5.95 ± 4.04	0.121
Benzhexol, *n* (%)^b^	32 (11.5%)	9 (11.7%)	23 (11.4%)	0.954
Amantadine, *n* (%)^b^	58 (20.9%)	17 (22.1%)	41 (20.4%)	0.758
Levodopa, *n* (%)^b^	216 (77.7%)	64 (83.1%)	152 (75.6%)	0.179
Dopamine agonists, *n* (%)^b^	148 (53.2%)	47 (61%)	101 (50.2%)	0.107
COMT inhibitors, *n* (%)^b^	37 (13.3%)	14 (18.2%)	23 (11.4%)	0.139
MAO-B inhibitors, *n* (%)^b^	54 (19.4%)	14 (18.2%)	40 (19.9%)	0.746
LEDs, mg^a^	484.78 ± 317.06	544.45 ± 330.59	461.93 ± 309.53	0.052
UPDRS-III^a^	27.08 ± 13.59	31.47 ± 15.20	25.40 ± 12.55	0.002^*^
HY, stage^a^	2.24 ± 0.81	2.53 ± 0.87	2.13 ± 0.77	< 0.001^*^

Variables are expressed as mean ± standard deviation (SD) or number (percentage). BMI: body mass index, COMT: Catechol-O-methyltransferase. HY stage, Hoehn and Yahr stage; LEDs, levodopa equivalent daily doses; MAO-B, Monoamine oxidase-B; UPDRS-III, the Unified Parkinson's Disease Rating Scale part III.

a Student's t-test.

b Chi-square test.

* Significant difference.

### Comparisons of non-motor symptoms between patients with and without hallucinations

The differences in non-motor symptoms between patients with and without hallucinations are shown in Table 3. After controlling for some variables, the patients with hallucinations were associated with significantly higher NMSQ (*P* < 0.001), HAMD (*P* = 0.005, *P* < 0.001), and HAMA (*P* = 0.002, *P* < 0.001) scores than the patients without hallucinations. In addition, hallucinations were associated with lower PDSS (*P* < 0.001) and MOCA scores (*P* < 0.001). These patients had worse cognitive performance and more severe sleep disturbances, depression, and anxiety.

**Table 3:** Non-motor characteristics of Parkinson's disease patients based on the presence/absence of hallucinations.

	**Total**	**With hallucinations**	**Without hallucinations**	***P***
NMSQ	11.45 ± 4.94	14.04 ± 4.83	10.45 ± 4.62	< 0.001^*^
HAMD	12.58 ± 8.88	15.04 ± 9.1	11.63 ± 8.63	0.005^*^
HAMA	10.4 ± 7.12	12.64 ± 6.82	9.54 ± 7.06	0.002^*^
PDSS	112.42 ± 19.77	104.64 ± 20.16	115.41 ± 18.83	< 0.001^*^
MOCA	24.81 ± 2.79	23.64 ± 2.43	25.26 ± 2.79	< 0.001^*^

Variables are expressed as mean ± standard deviation (SD). HAMA, Hamilton Anxiety Rating Scale; HAMD, Hamilton Depression Rating Scale; MOCA, Montreal Cognitive Assessment; NMSQ, Non-motor Symptoms Questionnaire; PDSS, PD Sleep Scale; PD, Parkinson's disease.

The *P*-value is calculated adjusted for age, UPDRS-III score, and HY stage.

* Significant difference.

### Comparisons of sleep parameters between patients with and without hallucinations

Approximately half of the patients (50.7%) in the cohort were diagnosed with sleep disorders (Table 4). Among the patients with sleep disorders, 53 (43.8%) reported hallucinations and 88 (68.8%) did not. In addition, we found a significant decrease in the TST, SE, REM sleep time, slow wave sleep (SWS, N3) time, and percentage and a corresponding increase in WASO and percentage of N2 sleep in PD patients with hallucinations. Moreover, the PLMI was higher in the hallucination group. However, light sleep (N1) time and percentage, MAI and AHI did not differ significantly between the two groups. Among patients with hallucinations (Supplementary Table S1), 45 (58.4%) experienced clear-cut hallucinations and 22 (28.6%) experienced hypnagogic hallucinations. Patients with clear-cut hallucinations had longer stage N1 sleep time (*p* = 0.007) and a higher percentage of N1 sleep (*p* = 0.003) than patients with hypnagogic hallucinations.

**Table 4:** Polysomnographic variables according to the presence/absence of hallucinations in Parkinson's disease patients.

	**Total**	**With hallucinations**	**Without hallucinations**	***P***
TST, min^b^	302.99 ± 93.40	253.02 ± 80.23	322.13 ± 91.14	< 0.001^*^
WASO, min^b^	173.86 ± 85.80	201.23 ± 89.29	163.38 ± 82.26	0.001^*^
SE, %^b^	57.35 ± 15.83	49.08 ± 14.04	60.51 ± 15.36	< 0.001^*^
SL, min^b^	34.21 ± 77.16	49.55 ± 133.17	28.33 ± 37.16	0.13
REML, min^a^	174.93 ± 110.77	178.71 ± 118.25	173.59 ± 108.3	0.741
REM, min^a^	42.39 ± 32.08	35.89 ± 30.95	44.90 ± 32.23	0.038^*^
REM, %^a^	14.02 ± 10.86	13.41 ± 10.26	14.26 ± 11.10	0.562
Stage N1, min^b^	30.21 ± 30.04	28.14 ± 30.48	31.00 ± 29.91	0.478
Stage N1, %b	10.39 ± 10.37	11.73 ± 13.17	9.88 ± 9.06	0.188
Stage N2, minb	179.53 ± 154.53	184.55 ± 269.40	177.60 ± 74.03	0.74
Stage N2, %b	56.51 ± 16.24	61.06 ± 17.62	54.77 ± 15.37	0.004^*^
Stage N3, minb	58.89 ± 53.47	34.37 ± 38.58	68.29 ± 55.45	< 0.001^*^
Stage N3, %b	19.99 ± 18.57	13.80 ± 13.48	22.35 ± 19.70	< 0.001^*^
MAI^b^	17.04 ± 16.08	16.29 ± 13.46	17.33 ± 16.99	0.63
AHI^b^	5.6 ± 9.35	6.41 ± 10.38	5.28 ± 8.93	0.368
PLMI^b^	22.53 ± 34.65	30.54 ± 44.21	19.47 ± 29.77	0.022^*^
With sleep disorders^c^ , *n* (%)	141(50.7%)	53(68.8%)	88(43.8%)	< 0.001^*^

Variables are expressed as mean ± standard deviation (SD).

AHI, apnea hypopnea index; MAI, micro-arousal index; PLMI, periodic limb movement index; REM, rapid eye movement; RSL, REM sleep latency; SE, sleep efficiency; SL, sleep latency; Stage N1, non-REM sleep stage 1; Stage N2, non-REM sleep stage 2; Stage N3, non-REM sleep stage 3; TST, total sleep time; WASO, wake time after sleep onset.

a The *P*-value is calculated adjusted for age.

b The *P*-value is calculated adjusted for age, UPDRS-III score, and HY stage.

c Chi-square test.

* Significant difference.

### Risk factors for hallucinations

Multivariate logistic regression analysis (Table 5) revealed that hallucinations were significantly related to the NMSQ score [OR: 1.17, 95% CI: (1.09–1.25)], MOCA score [OR: 0.82, 95% CI: (0.73–0.92)] and PSG variables such as sleep efficiency [OR: 0.95, 95% CI: (0.93–0.97)] and percentage of SWS out of the TST [OR: 0.96, 95% CI: (0.94–0.98)]. Hallucinators had more complex non-motor symptoms and more severe cognitive impairment. Improving sleep quality by elevating sleep efficiency and increasing deep sleep percentage may protect against hallucinations.

**Table 5:** Logistic regression analyses for independent predictors of hallucinations in Parkinson's disease patients.

	**OR**	**95% CI**	***P***
NMSQ	1.17	1.09–1.25	< 0.001
MOCA	0.82	0.73–0.92	0.001
SE%	0.95	0.93–0.97	< 0.001
N3%	0.96	0.94–0.98	0.001

CI, confidence interval; MOCA, Montreal Cognitive Assessment; N3, non-REM sleep stage 3; NMSQ, Non-motor Symptoms Questionnaire; OR, odds ratio; SE, sleep efficiency.

The *P*-value is calculated from a forward binary logistic regression analysis.

**Figure 1:**
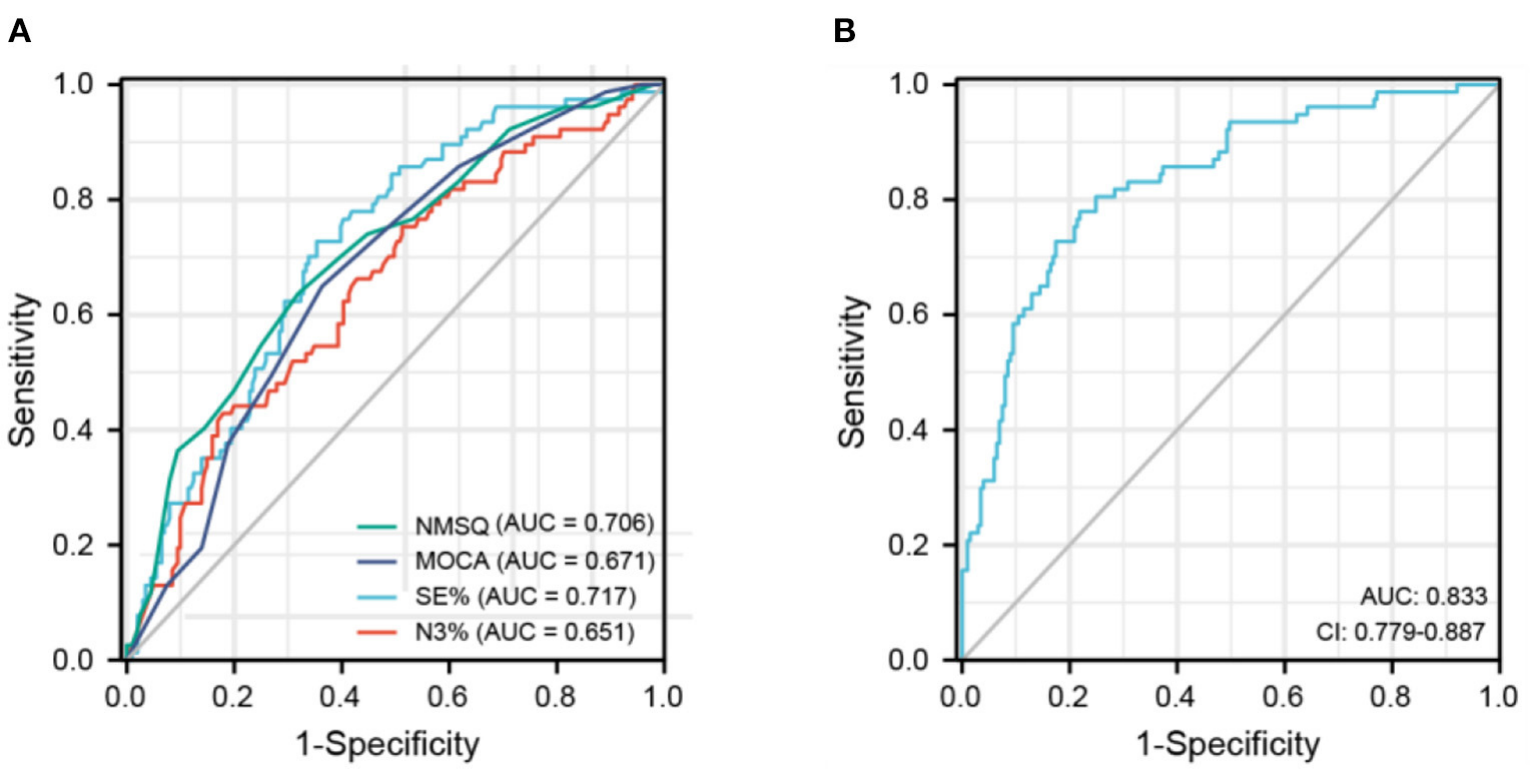
Receiver operating characteristic curve of predicting hallucinations in Parkinson's disease patients. **(A)** NMSQ score, MOCA score, SE%, N3% for independently predicting hallucinations occurrence in PD patients. **(B)** A combination of NMSQ score, MOCA score, SE%, N3% for predicting hallucinations occurrence in PD patients. AUC, area under the curve; MOCA, Montreal Cognitive Assessment; N3, non-REM sleep stage 3; NMSQ, Non-motor Symptoms Questionnaire; SE, sleep eciency.

A receiver operating characteristic (ROC) curve was used to assess the predictive value of the factors influencing hallucination occurrence (Figure 1). The NMSQ score, MOCA score, SE%, and N3% (Figure 1A) were used as independent indicators to plot the ROC curves, with the NMSQ score (AUC = 0.706, 95% CI: 0.638–0.774) and SE% (AUC = 0.717, 95% CI: 0.654–0.781) exhibiting good predictive value and the MOCA score (AUC = 0.671, 95% CI: 0.604–0.738) and N3% (AUC = 0.651, 95% CI: 0.579–0.723) exhibiting relatively less accurate predictive value. The ROC curve for the combined four indicators (Figure 1B) had a high sensitivity (77.9%) and specificity (78.1%) (AUC = 0.833: 95% CI: 0.779–0.887).

## Discussion

In previous cross-sectional studies, the prevalence of hallucinations in PD patients ranged from 21.5% to 50% (22–28). Our study reported that a quarter (25.8%) of PD patients experienced hallucinations. All patients with hallucinations experienced MHs or VHs, which were the most common modality of hallucination. As previously reported, PDP may occur in *de-novo* patients (5), MH can be observed in the earliest stages of the disease, followed by well-structured hallucinations, with gradual loss of insight and eventually progresses to delusions (29). Although the most hallucinatory content is not frightening, its development worried some PD patients. Hallucinations can be perceived as threatening when patients lose their insight (30).

Among our PD patients, the presence of hallucinations was significantly associated with older age, more severe motor and non-motor symptoms, such as HY stage, UPDRS-III score, depression, anxiety, impaired cognition and sleep disturbances. Multivariate analysis identified two clinical variables, the NMSQ score and MOCA score, as independent risk factors for hallucinations, supported by the results of similar studies (4, 27). Overall, as PD progressed, the motor symptoms became more severe and non-motor symptoms, including hallucinations and sleep disturbances, became more complex.

To the best of our knowledge, this is the first large sample study to explore the sleep architecture of Chinese PDP patients with PSG. The hallucinations group (68.8%) had a higher rate of sleep disorders than the non-hallucination group (43.8%). In addition, we found that patients with hallucinations exhibited less balanced and more fragmented sleep structure, with a corresponding reduction in the proportion of REM sleep and slow wave sleep. These findings support the hypothesis that REM sleep may intrude in the wakeful state and lead to visual hallucinations (31). Univariate analysis showed that the PDSS score was associated with hallucinations, but multivariate analysis revealed that it was not an independent predictor. We can determine that although questionnaire is convenient for assessing subjective sleep quality, it is less accurate than PSG measures.

Previous studies have reported a link between sleep disturbances and the occurrence of hallucinations. Healthy volunteers reported significant increases in paranoia and hallucinations after sleep loss (32). vConversely, treating volunteers with insomnia resulted in a reduction in paranoia and hallucinations (33). In the present study with PD patients, logistics regression analysis revealed that the SE% and N3% variables were independent predictors of hallucinations in PD patients. That is, reductions in sleep efficiency and deep sleep, which were found to lead to poor sleep quality, lethargy or fatigue the next day (34), may directly or indirectly exacerbate psychiatric problems in PD patients. First, sleep problems have functional impacts on perception and memory in PD patients (35, 36). The integrated theoretical hallucination model emphasizes dysregulation of gating and filtering exogenous perception and endogenous image creation; thus, aberrant release of stored percepts may be a way of compensating for reduced visual input, thus causing hallucinations (31, 37). Second, sleep is associated with neuroplasticity (38) and is essential for removing pathological brain proteins such as α-syn, amyloid-β, and tau proteins (39–41). In mice, sleep deprivation increased levels of tau protein in the interstitial fluid (ISF) almost 2-fold. Similar changes could be seen in the CSF of humans, as sleep deprivation significantly increased CSF α-syn, amyloid-β, and tau protein levels by 36.4, 30, and 51.5%, respectively. Chronic sleep disorders can interfere with the normal clearance process, leading to secondary pathological changes that may induce hallucinations in patients as the disease progresses. Finally, the development of hallucination (e.g., visual form) may occur in parallel to sleep problems throughout the course of PD (42), because the impaired neural structures affected by the disease are involved in sleep-wake control as well as visual perception (43). Although accumulating evidence strongly supports the interaction between sleep disturbances and hallucinations in PD patients, the underlying mechanisms of this bidirectional relationship remain unclear.

Although research on PD-related sleep problems has focused on the REM phase, NREM sleep, such as SWS, also plays a role in the pathogenesis of hallucinations (44). SWS is not only a major contributor to neurophysiological processes such as memory and attention, but it is also crucial for brain metabolite clearance (39, 45). Moreover, deeper sleep relates to slower motor progression and better cognitive performance in PD (46, 47).

Given the heterogeneity and complexity of PD and that combined indicators were more accurate than single indicators in predicting hallucinations in PD patients, we believe that various variables should be integrated to evaluate the condition of PD patients and predict the occurrence of hallucinations.

This study also has some limitations. For example, we monitored participant sleep for only one night, and there may have been a first-night effect. Moreover, hallucination symptoms and sleep conditions fluctuate; thus, measuring them at arbitrary time points does not provide a complete picture. Finally, our findings differed from previous findings due to a lack of specific hallucinatory content assessments and patient feedback. Future longitudinal cohort studies can assess the temporal association between psychosis and sleep disorders to determine whether sleep hygiene practices can alleviate sleep problems and hallucinations in PD patients.

In conclusion, approximately 1/4 of PD patients in our study experienced hallucinations. Over time, PD patients with sleep disturbances have an increased risk of psychotic hallucinations. Based on the PSG characteristics of PD patients with hallucinations in the present study, we propose that adjusting sleep behavior may control hallucinatory symptoms and improve the quality of life of individuals with PD.

## Data availability statement

The raw data supporting the conclusions of this article will be made available by the authors, without undue reservation.

## Ethics statement

The studies involving human participants were reviewed and approved by Ethics Committee of the Affiliated Brain Hospital of Nanjing Medical University. The patients/participants provided their written informed consent to participate in this study.

## Author contributions

LZ, JZ, and RG conceived and designed the study. LZ and JY obtained the funding. RG, JZ, MZ, SZ, YJ, YW, XJ, and BS collected the data. RG, JZ, and YP conducted the data analysis. JZ and RG drafted the manuscript. All authors contributed to the article and approved the submitted version.

## Funding

This work was funded by National Natural Science Foundation of China (Grant No. 82171249), Special Funds of the Jiangsu Provincial Key Research and Development Projects (Grant Nos. BE2018610 and BE2019612), Nanjing Rehabilitation Medicine Center Project, Jiangsu Provincial Cadre Health Projects (BJ20005), Jiangsu Provincial Elderly Health Research Project (LD2021013 and LR2021018), Nanjing Medical Science and Technology Development Foundation (QRX17026), and Nanjing Industrial and Information Development Special Fund Project.

## Conflict of interest

The authors declare that the research was conducted in the absence of any commercial or financial relationships that could be construed as a potential conflict of interest.

## Publisher's note

All claims expressed in this article are solely those of the authors and do not necessarily represent those of their affiliated organizations, or those of the publisher, the editors and the reviewers. Any product that may be evaluated in this article, or claim that may be made by its manufacturer, is not guaranteed or endorsed by the publisher.

## References

[1] Armstrong MJ Okun MS. Diagnosis and Treatment of Parkinson Disease: A Review. Jama. (2020) 323:548–60. 10.1001/jama.2019.22360 32044947

[2] Schapira AHV Chaudhuri KR Jenner P. Non-motor features of Parkinson disease. Nat Rev Neurosci. (2017) 18:435–50. 10.1038/nrn.2017.62 28592904

[3] Weerkamp NJ Tissingh G Poels PJ Zuidema SU Munneke M Koopmans RT. Non-motor symptoms in nursing home residents with Parkinson's disease: prevalence and effect on quality of life. J Am Geriatr Soc. (2013) 61:1714–21. 10.1111/jgs.12458 24117286

[4] Zhu K van Hilten JJ Putter H Marinus J. Risk factors for hallucinations in Parkinson's disease: results from a large prospective cohort study. Mov Disord. (2013) 28:755–62. 10.1002/mds.25389 23520046

[5] Lenka A Pagonabarraga J Pal PK Bejr-Kasem H Kulisvesky J. Minor hallucinations in Parkinson disease: a subtle symptom with major clinical implications. Neurology. (2019) 93:259–66. 10.1212/WNL.0000000000007913 31289146 PMC6709995

[6] Mahlknecht P Seppi K Poewe W. The concept of prodromal Parkinson's disease. J Parkinsons Dis. (2015) 5:681–97. 10.3233/JPD-150685 26485429 PMC4927924

[7] Kim YE Jeon BS. Clinical implication of REM sleep behavior disorder in Parkinson's disease. J Parkinsons Dis. (2014) 4:237–44. 10.3233/JPD-130293 24613864

[8] Barnes J Connelly V Wiggs L Boubert L Maravic K. Sleep patterns in Parkinson's disease patients with visual hallucinations. Int J Neurosci. (2010) 120:564–9. 10.3109/00207454.2010.494790 20615061

[9] Yu M Du Y Liu K Liang X Huang C He R. Sleep duration and auditory hallucinations: Genetic correlation and two-sample Mendelian randomization study. J Affect Disord. (2021) 291:409–14. 10.1016/j.jad.2021.04.038 33994199

[10] Lenka A Hegde S Jhunjhunwala KR Pal PK. Interactions of visual hallucinations, rapid eye movement sleep behavior disorder and cognitive impairment in Parkinson's disease: a review. Parkinsonism Relat Disord. (2016) 22:1–8. 10.1016/j.parkreldis.2015.11.018 26639978

[11] Postuma RB Berg D Stern M Poewe W Olanow CW Oertel W. MDS clinical diagnostic criteria for Parkinson's disease. Mov Disord. (2015) 30:1591–601. 10.1002/mds.26424 26474316

[12] Gill DJ Freshman A Blender JA Ravina B. The Montreal cognitive assessment as a screening tool for cognitive impairment in Parkinson's disease. Mov Disord. (2008) 23:1043–6. 10.1002/mds.22017 18381646

[13] Tomlinson CL Stowe R Patel S Rick C Gray R Clarke CE. Systematic review of levodopa dose equivalency reporting in Parkinson's disease. Mov Disord. (2010) 25:2649–53. 10.1002/mds.23429 21069833

[14] Chaudhuri KR Martinez-Martin P Schapira AH Stocchi F Sethi K Odin P. International multicenter pilot study of the first comprehensive self-completed nonmotor symptoms questionnaire for Parkinson's disease: the NMSQuest study. Mov Disord. (2006) 21:916–23. 10.1002/mds.20844 16547944

[15] Ciesielska N Sokołowski R Mazur E Podhorecka M Polak-Szabela A Kedziora-Kornatowska K. Is the Montreal Cognitive Assessment (MoCA) test better suited than the Mini-Mental State Examination (MMSE) in mild cognitive impairment (MCI) detection among people aged over 60? Meta-analysis. Psychiatr Pol. (2016) 50:1039–52. 10.12740/PP/45368 27992895

[16] Hamilton M. The assessment of anxiety states by rating. Br J Med Psychol. (1959) 32:50–5. 10.1111/j.2044-8341.1959.tb00467.x 13638508

[17] Williams JB. A structured interview guide for the Hamilton Depression Rating Scale. Arch Gen Psychiatry. (1988) 45:742–7. 10.1001/archpsyc.1988.01800320058007 3395203

[18] Chaudhuri KR Pal S DiMarco A Whately-Smith C Bridgman K Mathew R. The Parkinson's disease sleep scale: a new instrument for assessing sleep and nocturnal disability in Parkinson's disease. J Neurol Neurosurg Psychiatry. (2002) 73:629–35. 10.1136/jnnp.73.6.629 12438461 PMC1757333

[19] Zhu J Shen B Lu L Lan W Pan Y Zhang L. Prevalence and risk factors for visual hallucinations in Chinese patients with Parkinson's disease. J Neurol Sci. (2017) 372:471–6. 10.1016/j.jns.2016.10.043 27823833

[20] Berry RB Budhiraja R Gottlieb DJ Gozal D Iber C Kapur VK. Rules for scoring respiratory events in sleep: update of the 2007 AASM manual for the scoring of sleep and associated events. Deliberations of the sleep apnea definitions task force of the American academy of sleep medicine. J Clin Sleep Med. (2012) 8:597–619. 10.5664/jcsm.2172 23066376 PMC3459210

[21] Zhu J Zhong M Yan J Jiang X Wu Z Pan Y. Non-motor symptoms affect sleep quality in early-stage parkinson's disease patients with or without cognitive dysfunction. Front Neurol. (2020) 11:292. 10.3389/fneur.2020.00292 32373056 PMC7186472

[22] Lee AH Weintraub D. Psychosis in Parkinson's disease without dementia: common and comorbid with other non-motor symptoms. Mov Disord. (2012) 27:858–63. 10.1002/mds.25003 22674352 PMC3511789

[23] Barrett MJ Smolkin ME Flanigan JL Shah BB Harrison MB Sperling SA. Characteristics, correlates, and assessment of psychosis in Parkinson disease without dementia. Parkinsonism Relat Disord. (2017) 43:56–60. 10.1016/j.parkreldis.2017.07.011 28735797

[24] Ffytche DH Pereira JB Ballard C Chaudhuri KR Weintraub D Aarsland D. Risk factors for early psychosis in PD: insights from the Parkinson's progression markers initiative. J Neurol Neurosurg Psychiatry. (2017) 88:325–31. 10.1136/jnnp-2016-314832 28315846 PMC5362125

[25] Mack J Rabins P Anderson K Goldstein S Grill S Hirsch ES. Prevalence of psychotic symptoms in a community-based Parkinson disease sample. Am J Geriatr Psychiatry. (2012) 20:123–32. 10.1097/JGP.0b013e31821f1b41 21617521 PMC3168582

[26] Gibson G Mottram PG Burn DJ Hindle JV Landau S Samuel M. Frequency, prevalence, incidence and risk factors associated with visual hallucinations in a sample of patients with Parkinson's disease: a longitudinal 4-year study. Int J Geriatr Psychiatry. (2013) 28:626–31. 10.1002/gps.3869 22927195

[27] Zhang Y Zhang GY Zhu XB Zhang ZE Gan J Liu ZG. Clinical characteristics of minor hallucinations in Chinese Parkinson's disease patients. Front Aging Neurosci. (2021) 13:723405. 10.3389/fnagi.2021.723405 35126085 PMC8810481

[28] Omoto S Murakami H Shiraishi T Bono K Umehara T Iguchi Y. Risk factors for minor hallucinations in Parkinson's disease. Acta Neurol Scand. (2021) 143:538–44. 10.1111/ane.13380 33222164

[29] Ffytche DH Creese B Politis M Chaudhuri KR Weintraub D Ballard C. The psychosis spectrum in Parkinson disease. Nat Rev Neurol. (2017) 13:81–95. 10.1038/nrneurol.2016.200 28106066 PMC5656278

[30] Marinus J Zhu K Marras C Aarsland D van Hilten JJ. Risk factors for non-motor symptoms in Parkinson's disease. Lancet Neurol. (2018) 17:559–68. 10.1016/S1474-4422(18)30127-3 29699914

[31] Muller AJ Shine JM Halliday GM Lewis SJ. Visual hallucinations in Parkinson's disease: theoretical models. Mov Disord. (2014) 29:1591–8. 10.1002/mds.26004 25154807

[32] Reeve S Emsley R Sheaves B Freeman D. Disrupting sleep: the effects of sleep loss on psychotic experiences tested in an experimental study with mediation analysis. Schizophr Bull. (2018) 44:662–71. 10.1093/schbul/sbx103 28981834 PMC5890488

[33] Freeman D Sheaves B Goodwin GM Yu LM Nickless A Harrison PJ. The effects of improving sleep on mental health (OASIS): a randomised controlled trial with mediation analysis. Lancet Psychiatry. (2017) 4:749–58. 10.1016/S2215-0366(17)30328-0 28888927 PMC5614772

[34] Yin Z Bai Y Guan B Jiang Y Wang Z Meng F. A quantitative analysis of the effect of bilateral subthalamic nucleus-deep brain stimulation on subjective and objective sleep parameters in Parkinson's disease. Sleep Med. (2021) 79:195–204. 10.1016/j.sleep.2020.10.021 33208282

[35] Kirszenblat L Ertekin D Goodsell J Zhou Y Shaw PJ van Swinderen B. Sleep regulates visual selective attention in Drosophila. J Exp Biol. (2018) 221:jeb191429. 10.1242/jeb.191429 30355611 PMC6307875

[36] Klinzing JG Niethard N Born J. Mechanisms of systems memory consolidation during sleep. Nat Neurosci. (2019) 22:1598–610. 10.1038/s41593-019-0467-3 31451802

[37] Diederich NJ Goetz CG Stebbins GT. Repeated visual hallucinations in Parkinson's disease as disturbed external/internal perceptions: focused review and a new integrative model. Mov Disord. (2005) 20:130–40. 10.1002/mds.20308 15486924

[38] Tononi G Cirelli C. Sleep and the price of plasticity: from synaptic and cellular homeostasis to memory consolidation and integration. Neuron. (2014) 81:12–34. 10.1016/j.neuron.2013.12.025 24411729 PMC3921176

[39] Ju YS Ooms SJ Sutphen C Macauley SL Zangrilli MA Jerome G. Slow wave sleep disruption increases cerebrospinal fluid amyloid-β levels. Brain. (2017) 140:2104–11. 10.1093/brain/awx148 28899014 PMC5790144

[40] Wang C Holtzman DM. Bidirectional relationship between sleep and Alzheimer's disease: role of amyloid, tau, and other factors. Neuropsychopharmacology. (2020) 45:104–20. 10.1038/s41386-019-0478-5 31408876 PMC6879647

[41] Holth JK Fritschi SK Wang C Pedersen NP Cirrito JR Mahan TE. The sleep-wake cycle regulates brain interstitial fluid tau in mice and CSF tau in humans. Science. (2019) 363:880–4. 10.1126/science.aav2546 30679382 PMC6410369

[42] Zahed H Zuzuarregui JRP Gilron R Denison T Starr PA Little S. The neurophysiology of sleep in Parkinson's disease. Mov Disord. (2021) 36:1526–42. 10.1002/mds.28562 33826171

[43] Damulewicz M Ispizua JI Ceriani MF Pyza EM. Communication among photoreceptors and the central clock affects sleep profile. Front Physiol. (2020) 11:993. 10.3389/fphys.2020.00993 32848895 PMC7431659

[44] Manni R. Rapid eye movement sleep, non-rapid eye movement sleep, dreams, and hallucinations. Curr Psychiatry Rep. (2005) 7:196–200. 10.1007/s11920-005-0053-0 15935133

[45] Léger D Debellemaniere E Rabat A Bayon V Benchenane K Chennaoui M. Slow-wave sleep: from the cell to the clinic. Sleep Med Rev. (2018) 41:113–32. 10.1016/j.smrv.2018.01.008 29490885

[46] Schreiner SJ Imbach LL Werth E Poryazova R Baumann-Vogel H Valko PO. Slow-wave sleep and motor progression in Parkinson disease. Ann Neurol. (2019) 85:765–70. 10.1002/ana.25459 30887557

[47] Wood KH Memon AA Memon RA Joop A Pilkington J Catiul C. Slow wave sleep and EEG delta spectral power are associated with cognitive function in Parkinson's disease. J Parkinsons Dis. (2021) 11:703–14. 10.3233/JPD-202215 33361608 PMC8058231

