# Supplementary figures and images for: Characteristics of sleep structure in Parkinson's disease patients with hallucinations based on polysomnography
Source: Front Neurol. 2022 Nov 1;13:929569. doi: 10.3389/fneur.2022.929569 (PMC9663659; doi:10.3389/fneur.2022.929569)

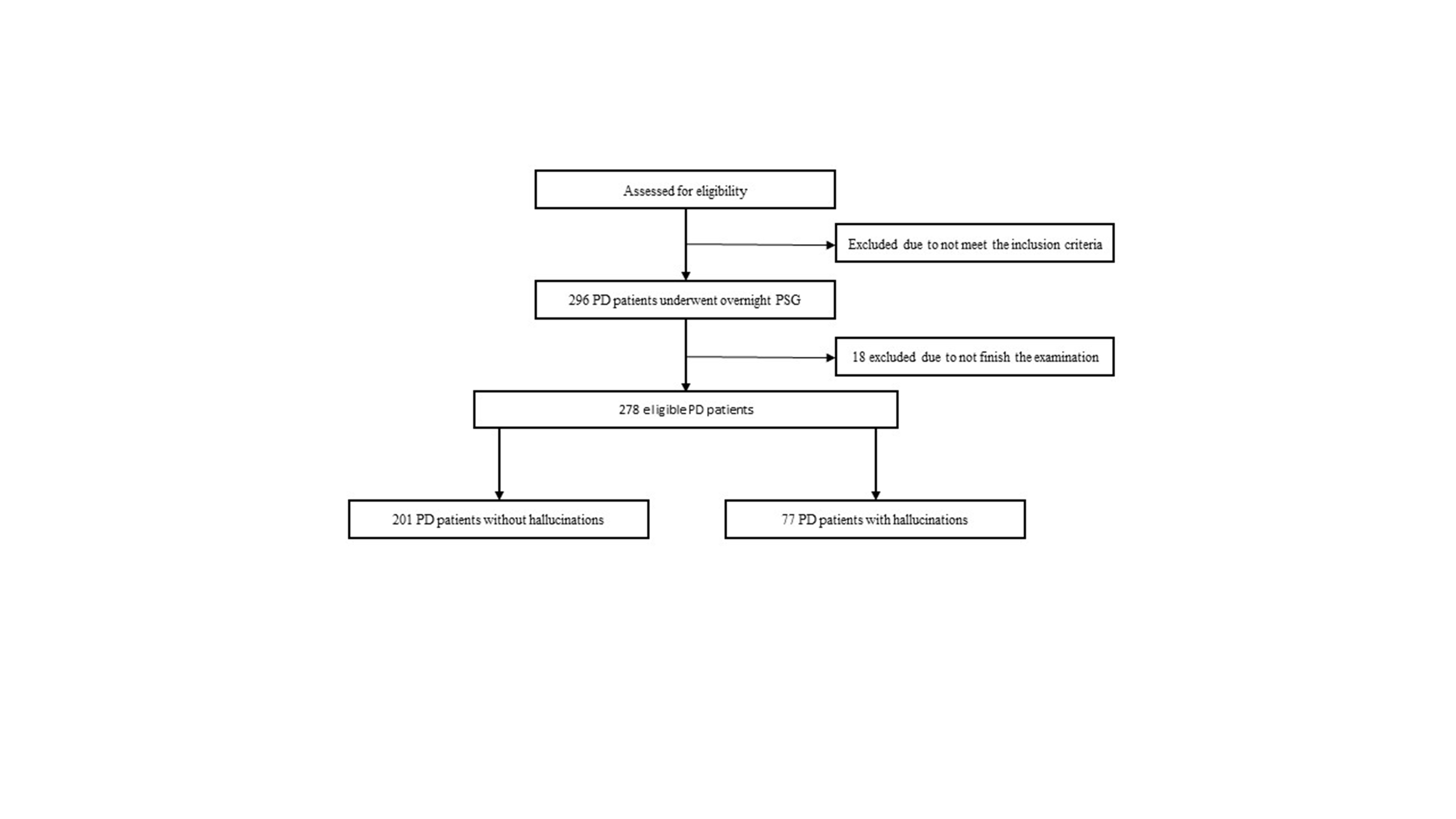

Supplement: Supplementary file 1 [file Image_1.jpeg]
