# Supplementary material for: Characteristics of sleep structure in Parkinson's disease patients with hallucinations based on polysomnography
Source: Front Neurol. 2022 Nov 1;13:929569. doi: 10.3389/fneur.2022.929569 (PMC9663659; doi:10.3389/fneur.2022.929569)
Supplement: Supplementary file 2 [file Table_1.docx]

**Table S1. Comparisons of clinical characteristics and PSG parameters between clear-cut hallucinations group and hypnagogic hallucinations group.**

|  | Clear-cut hallucinations | Hypnagogic hallucinations | P |
| --- | --- | --- | --- |
| n | 45(58.4%) | 22(28.6%) |  |
| Age, y | 67.78±7.3 | 69.59±9.78 | 0.446 |
| LEDs, mg | 522.98±332.69 | 557.98±355.56 | 0.694 |
| UPDRS-III | 32.42±15.87 | 32.14±16.06 | 0.945 |
| HY, stage | 2.58±0.95 | 2.57±0.76 | 0.967 |
| NMSQ | 14.69±4.53 | 12.86±5.6 | 0.192 |
| HAMD | 15.91±9.21 | 14.32±10.02 | 0.521 |
| HAMA | 13±7.39 | 12.23±5.71 | 0.668 |
| PDSS | 102.2±19.49 | 108.45±22.44 | 0.245 |
| MOCA | 23.82±2.46 | 23.41±2.46 | 0.521 |
| TST, min | 260.33±79.75 | 250.59±82.43 | 0.644 |
| WASO, min | 200.83±88.11 | 195.07±91.7 | 0.805 |
| SE, % | 51.42±14.11 | 46.33±14.01 | 0.170 |
| SL, min | 52.7±169.59 | 56.52±58.06 | 0.919 |
| REML, min | 160.26±95.49 | 171.33±119.44 | 0.706 |
| REM, min | 35.26±30.09 | 38.39±31.6 | 0.695 |
| REM, % | 12.96±10.02 | 14.33±11.29 | 0.617 |
| Stage N1, min | 33.63±34.16 | 15.5±18.91 | 0.007* |
| Stage N1, % | 13.85±14.81 | 5.98±5.89 | 0.003* |
| Stage N2, min | 160.16±72.15 | 261.59±490.83 | 0.346 |
| Stage N2, % | 60.68±18.15 | 65.16±17.38 | 0.340 |
| Stage N3, min | 33.54±42.68 | 35.25±31.47 | 0.868 |
| Stage N3, % | 12.52±12.69 | 14.5±12.37 | 0.548 |
| MAI | 15.86±12.11 | 18.66±17.31 | 0.445 |
| AHI | 5.83±9.7 | 6±10.3 | 0.947 |
| PLMI | 36.31±53.57 | 19.21±21.08 | 0.067 |

Variables are expressed as mean ± standard deviation (SD) or number (percentage).

AHI: apnea hypopnea index, HAMA: Hamilton Anxiety Rating Scale, HAMD: Hamilton Depression Rating Scale, MAI: micro-arousal index, MOCA Montreal Cognitive Assessment, NMSQ: Nonmotor Symptoms Questionnaire, PDSS: PD Sleep Scale, PLMI: periodic limb movement index, REM: rapid eye movement, RSL: REM sleep latency, SE: sleep efficiency, SL: sleep latency, Stage N1: non-REM sleep stage 1, Stage N2: non-REM sleep stage 2, Stage N3: non-REM sleep stage 3, TST: total sleep time, WASO: wake time after sleep onset.

The P value is calculated by Student's t-test

*Significant difference.
